# Geometrid caterpillar in Eocene Baltic amber (Lepidoptera, Geometridae)

**DOI:** 10.1038/s41598-019-53734-w

**Published:** 2019-11-20

**Authors:** Thilo C. Fischer, Artur Michalski, Axel Hausmann

**Affiliations:** 1 Thilo C. Fischer, SNSB - Bavarian State Collection of Zoology, Münchhausenstraße 21, D-81247 Munich, Germany; 2 Artur Michalski, private investigator, Ul.Mielczarskiego 2/1, 51-663 Wrocław, Polska Poland; 3 Axel Hausmann, SNSB - Bavarian State Collection of Zoology, Münchhausenstraße 21, D-81247 Munich, Germany

**Keywords:** Evolutionary developmental biology, Palaeontology

## Abstract

Lepidoptera have little fossilization potential due to the presence of delicate structures and hence are exceptional findings, even in ambers that allow their preservation in sufficient detail for interpretation. From Eocene Baltic amber, the volumetrically largest known deposit of amber, there has been no reliable report of any member of the Macrolepidoptera (informal group of higher moths and all butterflies). Any such lepidopteran fossil would provide insight into evolutionary processes during the Eocene, long after flowering plants had completed their initial radiation. Here, we report on a first geometrid caterpillar from Baltic amber which is described as the oldest evidence for the subfamily Ennominae (tribe Boarmiini) and as one of the oldest records of the currently mega-diverse family. The new finding provides an important calibration point for molecular clock analyses within the family Geometridae and predates the basal divergence of Boarmiini from 32–38 to 44 Mya. It also predates the occurrence of this highly specialized form of caterpillar locomotion that allows for rapid movement.

## Introduction

Fossil Lepidoptera are among the rarest findings among insect orders, and mainly occur in ambers. The family Geometridae presently is unknown from Baltic amber^1,2^, the main deposit of amber. Their caterpillars are familiar as geometers, inchworms or loopers due to their peculiar way of moving, accompanied by a reduced number of prolegs. Molecular phylogenetic and fossil evidence indicate that the diversification of Geometridae is dated at 54 mya (62-48 mya (million years ago)), corresponding to Middle Paleocene to Lower Eocene^3–6^. The geologic age of Baltic amber is given as Mid Eocene (Lutetium) (44 Mya)^7^.

Geometridae is a family of macrolepidopterans within the superfamily Geometroidea^8^. The prolegs of their caterpillars are reduced as a functional adaptation to the specialized locomotion of the caterpillars, leaving only the anal prolegs at abdominal segment A10 and another pair at A6^9,10^. Geometridae is one of the three largest families of Lepidoptera, comprising 23,000 valid species in 8 subfamilies, that contain 2005 genera^11,12^. Geometridae caterpillars live mostly on trees and shrubs^10^. A first fossil geometrid was described based on an adult forewing from the sediments of Florissant Formation in Colorado, USA^2,3^. Fossilized loopers in amber presently had only been known from Miocene amber of the Dominican Republic (16 mya)^13^.

## Results

### Authentication of the amber

The caterpillar inclusion shows a typical cloudiness on one side of the specimen which occurs preferentially in Baltic amber and other succinites like from Bitterfeld, Germany, or Rovno, Ukraine. The effect is from the embedding of the inclusions with sufficient moisture that a microemulsion of water is created in the resin, forming only in the shadow of the inclusion. Such an effect is difficult to copy with artificial resins and extant insects. Most importantly, the amber was obtained from a local amber seller known to author AM, being an established and trusted source.

### Eogeometer vadens nov. gen., nov. spec

Systematics according to van Nieukerken *et al*.^8^

Order LEPIDOPTERA Linnaeus, 1758

Clade DITRYSIA Börner, 1925

Superfamily GEOMETROIDEA Leach, 1815

Family GEOMETRIDAE Leach, 1815

Genus *Eogeometer* nov. gen.

Etymology: The prefix ‘Eo’ refers to the Eocene, ‘geometer’ to the family name.

Species *Eogeometer vadens* nov. spec.

**Figure 1:**
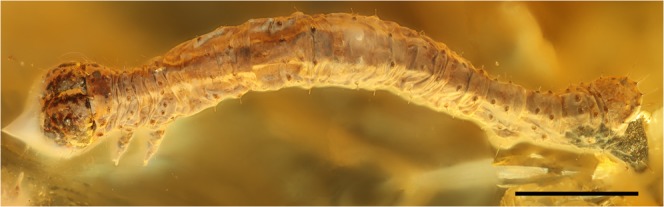
Looping geometrid caterpillar from Baltic amber. It is situated in a dorsal to lateral aspect with the Geometridae-type prolegs clearly visible. Scale bar equals 1 mm. The software Helicon Focus Mac 7.0.1 was used (https://www.heliconsoft.com/helicon-focus-history-of-changes-mac/).

Holotypus: Specimen SNSB-ZSM-LEP amb002 (Fig. 1)

Locus typicus: Amber mine of Yantarni, Russia

Stratum typicum: “Blaue Erde” Horizon (Upper Eocene – Lower Oligocene)

Etymology: The species name ‘*vadens*’ - “walking” refers to the looping manner of locomotion.

Repository: Bavarian State Collection of Zoology, Munich, Germany; accession number SNSB-ZSM-LEP amb002.

#### Diagnosis of genus

The genus *Eogeometer* is established for this Eocene geometrid caterpillar presumably belonging to the tribe Boarmiini. A differential diagnosis will be given when more Eocene geometrids will become known. Attribution to any extant geometrid genus would be highly speculative. The amber larva shares the character combination of supposed conifer-feeding (see below) and a conspicuous semicircular anal plate with only a few extant geometrid larvae (see discussion), which, however, do not show a longitudinal stripe pattern.

#### Description of species

The looping caterpillar is 5 mm in length; the body is long and approximately cylindrical, smooth, but with transversal wrinkles. Along the uppermost ventral side of thorax and abdomen runs a longitudinal protruding and darkish stripe. The head diameter is about 1.5 times the diameter of the body, hypognathous and rounded; both parts of the head capsule show a symmetric spotty dark and light pattern. The legs are well developed (first right leg is missing); prolegs are well developed at abdominal segments A6 and A10 (one proleg of A10 is broken off and is preserved at some distance to the head of the caterpillar); only rudimentary prolegs are present at abdominal segment 5. There is a shield-like protrusion from the ventral side of A10. The setae D1, D2 are positioned at the edge of the longitudinal, dark protrusion. L1, L2 and SV1 are present in lateral positions. MD1, MSD1, MSD2, SD1, SD2 are seemingly absent (Setal map as Supplementary Information).

#### Taphonomy

The geometrid inclusion occurs within a flat piece of non-autoclaved Baltic amber with dimensions 30 mm by 20 mm, and a height of 4 mm. At one edge, about 5 mm were removed from the sample for IR analysis. The caterpillar is situated centrally within the amber, close to an internal boundary of two resin flows. The only syninclusions preserved adjacent the caterpillar are two different but poorly preserved mites. Some pyrite is present in cracks of the amber.

## Discussion

### Systematic identification

The specimen shows the characteristics of Geometridae caterpillars. The most important features of loopers are the presence of strongly developed prolegs only at abdominal segments A6 and A10, but “sometimes with small or rudimentary prolegs on abdominal prolegs 4 and 5”^14–16^. A rudimentary pair of prolegs at A5 is found in this case, which conforms with an identification as Geometridae, where prolegs of A5 are always much smaller than those of A6^15^, while in other semi-looping caterpillars (e.g. Erebidae, Plusiinae etc.) the prolegs on (A4-)A5 are fully developed. A longitudinal (dark and protruding) stripe runs along the length of the body and an eminence is present (at A10). These traits are given by Scoble^9^ for Geometridae. The claws of the thoracic legs are straight and stretched, not strongly curved, the latter being a plesiomorphic feature of some geometrids^15^.

The caterpillar represents a first or second instar, as indicated by the incomplete development of setae (reduced number of lateral setae on abdominal segments). Further indications for an early instar are the small size for a geometrid caterpillar, and its round head^10^. The large relative width of the head compared with the body width as well as the conspicuous anal plate^9,16^ are likely related to recent moulting; hence, the larva is very likely to be in the early L2 stage. In contrast, a broader head capsule is present because larger mandibles were stated as a characteristic of conifer-feeding *Ectropis crepuscularia* (tribe Boarmiini) by Sato^17,18^ and Sommerer^19^.

The amber larva shows similarities (character combination of semicircular anal plate with posterior setae, warts at the base of setae, position and shape of legs, absence of other processes) with young larval instars of extant species of the genera *Alcis* (e.g. *A. jubata*), *Hypomecis* and *Peribatodes*. Consequently the specimen likely belongs to the tribus Boarmiini subfamily Ennominae. Mesoseries at the crochets of the prolegs, a characteristic of macrolepidoptera, are generally not visible due to non-transparent amber regions at the ventral side of the specimen. The additional (vestigial) proleg on A5 excludes membership in one of the other larger subfamilies of Geometrinae, Sterrhinae, and Larentiinae^15^.

### Taphonomy

Evident injuries on the specimen (loss of a leg) are obviously due to the process of embedment by the resin flow which knocked the looper off from its support. There is silk produced from the spinneret, which itself is hidden behind this silk and some non-transparent amber around it.

The caterpillar is found in a typical looping position. This position may have been the result of being entombed during the very moment of movement and resin contact. Alternatively, the position has been a reaction on the resin contact itself, inducing an active looper position as part of an escape reaction. The latter interpretation seems much more likely; the disruption of (pro)legs by the resin flow probably ensures that a passive embedment in a living position is a less likely interpretation.

### Habitat and feeding

The finding of a caterpillar which is less mobile than its conspecific lepidopteran adults indicates that the coniferous resin’s mother plant was the most likely habitat of the preimaginal stage of *Eogeometer vadens*. While most extant geometrids feed on angiosperm trees and shrubs, a few genera and species live on conifers or lichens and thus would be more naturally associated to the resin producing host trees in the amber forests. Interestingly some of the above-mentioned genera with similar larvae such as *Alcis* and *Peribatodes* include extant species associated with conifers or lichens^20^. Conifer- and lichen-feeding is a widespread phenomenon present in other extant boarmiine genera, including *Tephronia*, *Bupalus, Fagivorina, Deileptenia, Hypomecis, Ectropis, Ekboarmia, Afriberina*, and *Ecleora*^20^. The longitudinally striped pattern of *Eogeometer vadens* might be a camouflage among gymnosperm needles.

### Relation to other fossil findings of Geometridae

The basal divergence of the major Macrolepidoptera lineages has been postulated for the Upper Cretaceous. The superfamily Geometroidea was present during the Paleocene/Eocene^1,6^. The only previous report of a macrolepidopteran inclusion in Baltic amber is a dubious *Sphinx*^21^, which was later revised as Lepidoptera indet^2,22^.

An interesting question is why Geometridae are so rare in Baltic amber? The family currently is highly diverse and occupies several habitats, mostly in forest ecosystems. Larvae of extant Geometridae typically feed on trees and shrubs, in contrast to the herbaceous hosts of many other macrolepidopterans^1,10^. Macrolepidopteran imagos should be rare as inclusions in ambers simply due to their size. However, this is not necessarily valid for their caterpillars, especially for the earlier and smaller instars.

Possibly, geometrid caterpillars living in Baltic amber forests are underrepresented in amber due to their nocturnal activity whilst resin is fluid mainly during higher temperatures at day and direct sunlight.

### Calibration points for molecular clocks

Dated phylogenies involving molecular clock approaches essentially need to be based on reliably dated and identified calibration points. Unfortunately, for the family Geometridae only a few fossils are known, most with questionable and highly speculative attribution to higher taxa such as genus, tribe or subfamily^2^. Presently only the Florissant larentiine moth (forewing) described as ‘*Hydriomena protrita* Cockerell, 1922’ -but probably misplaced in the extant genus *Hydriomena*- provides a valuable calibration point supporting a minimum divergence of 41 Mya for the subfamily Larentiinae. *Geometridites larentiiformis*^23^ from late Eocene of the United Kingdom as well seems to be a larentiine moth, judged from the double areole on the forewing. Considerably younger fossils from the Upper Miocene (approx. 8 Mya) such as the geometrid *Problongos baudiliensis* Merit & Merit, 2008, identified as member of the subfamily Ennominae^24^, are less suitable for fixing minimum divergences in geometrid phylogeny. In current molecular clock phylogenies, the basal divergences at subfamily level in Geometridae are roughly dated to the time interval between 42 and 54 Mya or 70 Mya^5,25,26^, and the earliest divergences within the Ennominae to about 35–41 Mya^5,25,26^. Given the present results of *Eogeometer* we can fix a calibration point to approx. 44 Mya for the minimum basal divergence of Ennominae which somewhat predates the previously assumed divergence dates. However, when considering the (quite likely) identity of our larva as a boarmiine moth, the minimum basal branching for this tribe needs to be corrected from the previous 32–38 Mya^5,25,26^ to the current 44 Mya. These new dating results do not affect the estimate of the minimum age of the geometrid family and thus have no influence on the age estimates of other lepidopteran families, however, they will lead to a slight pre-dating of all other geometrid subfamilies (cf. current hypothesis on the geometrid phylogeny^27^).

## Methods

The specimen was preserved by treatment with an acryl varnish and is kept at constant temperature in plastic clip bags within metal boxes, to exclude oxygen and light. It will be deposited at the SNSB – Bavarian State Collection of Zoology, Munich with the number SNSB-ZSM-LEP amb002. Due to the presence of pyrite the inclusion could not be studied by micro-computer-tomography. Photographs were taken with a digital imaging system consisting of a Canon 5DS camera with, and 10x ELWD Plan Apo objectives attached to a Carl Zeiss Jena Sonnar 3.5 / 135 MC as focus lens. Illumination was with two Canon Speedlite 430EX III-RT flashlights and translucent paper diffusors. Image stacks were generated using the Stackmaster macro rail (Stonemaster), and images were then assembled with the computer software Helicon Focus Mac 7.0.1.

## Supplementary information

Supplementary Material
