# Supplementary material for: Geometrid caterpillar in Eocene Baltic amber (Lepidoptera, Geometridae)
Source: Sci Rep. 2019 Nov 20;9:17201. doi: 10.1038/s41598-019-53734-w (PMC6868187; doi:10.1038/s41598-019-53734-w)
Supplement: Supplementary file 1 — Supplementary Material [file 41598_2019_53734_MOESM1_ESM.docx]

**Geometrid caterpillar in Eocene Baltic amber (Lepidoptera, Geometridae)**

Thilo C. Fischer, Artur Michalski, Axel Hausmann

**Supplementary information**


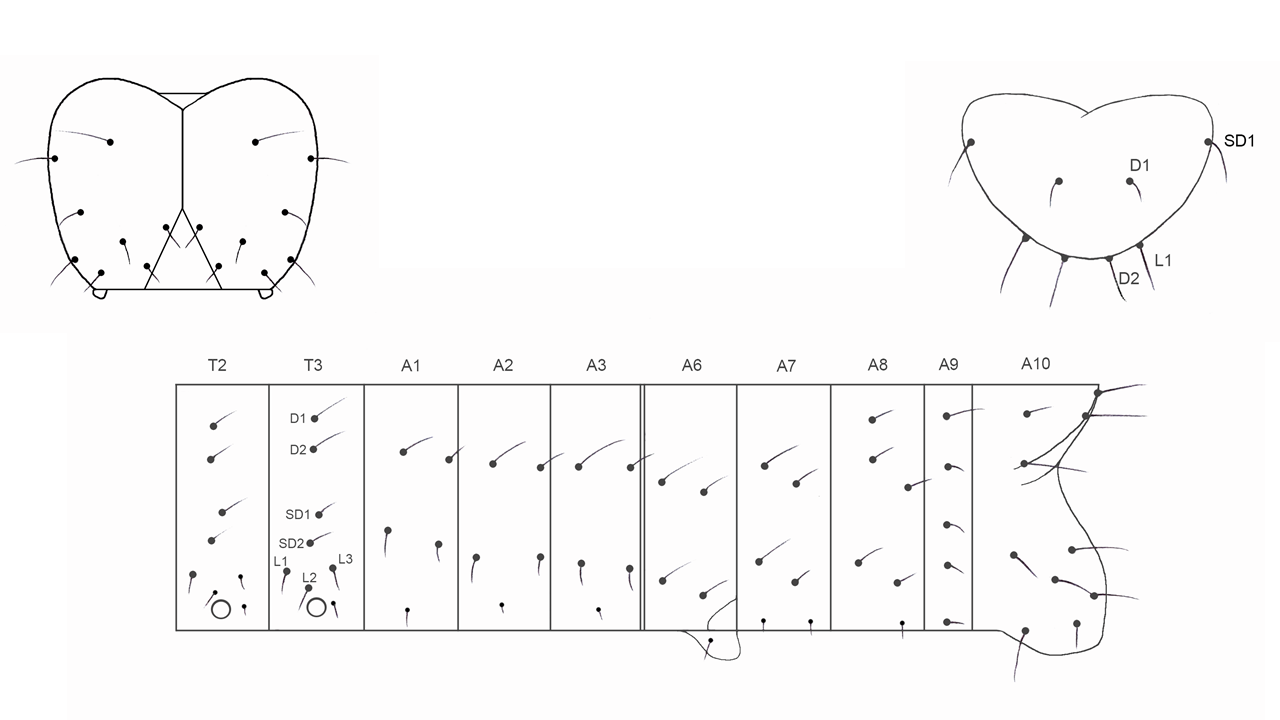


Supplementary Figure 1: Setal map of geometrid caterpillar from Baltic amber. The setal maps show the setae mentioned in the text in a standard format.
